# Board of Regent’s Request

**Published:** 1895-04

**Authors:** 


IN compliance with the request of the Board of Regents the entire homeopathic faculty of the University of Michigan have tendered their resignations, to take effect at the close of the present college year.

